# Ecosystem guidance for the incorporation of renewable utilities in a multi-use campus network

**DOI:** 10.1371/journal.pone.0267431

**Published:** 2022-05-19

**Authors:** Shelby Warrington, Astrid Layton

**Affiliations:** 1 Yale School of the Environment, Yale University, New Haven, CT, United States of America; 2 J. Mike Walker 66’ Department of Mechanical Engineering, Texas A&M University, College Station, TX, United States of America; National Institute of Technology Silchar, India, INDIA

## Abstract

Configuring the network connections in industrial, power, and water networks to mimic the structural patterns of ecological food webs has been shown to improve the resilience of human networks. This work investigates the ability of food web inspiration to specifically guide the incorporation of renewable energy and water sources for resilience. Feasibility is tested using the water and electricity networks of the Texas A&M University main campus, demonstrating the potential of university campus case studies as analogies for other multi-use networks, such as cities or industrial-commercial regions, due to the variety of functions met within the system boundaries. Ecological robustness, the unique and characteristic behavior of ecosystems to slightly favor redundancy over efficiency, is used to correlate the incorporation and supply-levels of solar power and rainwater collection in a realistic campus model with the overall resilience of the electricity and domestic water networks. Non-obviously, the results suggest that the ecologically-similar resilience is achieved when less than 100% of utilities come from renewable sources, indicating an important potential tradeoff between efforts to shift to 100% renewable sources and network resilience concerns.

## Introduction

### Sustainable systems

Growing interest in the creation of sustainable systems has led to the creation of multiple strategies to combat problems of carbon emissions, waste generation, linear product life cycles, water scarcity, and other sustainability issues. One example of this is circular economy (CE), a strategy to increase the efficiency of resource use by “closing-the-loop” between raw materials used in production and the waste created post-consumption [1]. A subset of circular economy is industrial symbiosis (IS), when industries exchange by-products to replace a material import with what would have been a wasted export—reducing both raw material use and waste production [2–4]. Past applications have shown that industrial symbiosis has the potential to maximize the output of systems while minimizing waste [5–7]. Other benefits from IS include overall cost-savings and mitigation of greenhouse gases [8, 9]. Clusters of industries with mutually beneficial interactions are known as an eco-industrial network. When the industries are within the boundaries of an industrial park they are more specifically known as an eco-industrial park (EIP). EIPs have received a lot of attention for their ability to successfully match waste streams with needed raw materials [10–13]. The Kalundborg EIP in Kalundborg, Denmark for example began forming in 1961 in response to a lack of groundwater [14–16]. Since the initial water sharing connections, over 30 additional material and energy ‘mutually beneficial’ interactions have been added to the network—leading to reductions in annual carbon emissions, landfill contributions and material imports [16–19]. The success of Kalundborg however has not been easily translated to the ground up design of EIPs, suggesting that for the industrial symbiosis of EIPs to be successful the symbiotic relationships cannot be forced–they must organically develop over time. Industrial symbiosis relies heavily on cooperation and information sharing between actors, which can be difficult to achieve when industries have proprietary information they are not willing to share [20]. Other barriers to the adoption of IS strategies are regulations that discourage waste reuse and an unwillingness to sacrifice short-term economic returns [20].

Net-zero communities (NZCs) are another strategy aimed at improving sustainability at a network level. These networks include residential and urban areas and are designed towards goals such as zero net energy consumption and net zero carbon emissions. Unlike net-zero buildings, the design of net-zero communities involves consideration of interactions between various building and energy sources within the community [21]. While net-zero communities can potentially bring about advantages that reduce costs over time, differences in the density and scale of a community can greatly affect the potential costs. In some cases, building level solutions are actually more cost effective than community level solutions [21]. The design/planning stage also plays an important role in the development of net-zero communities, which may make it necessary to create net zero communities from scratch as opposed to changing existing networks [21]. Current net zero community efforts are few and remain experimental [22, 23]. One potential avenue to circumvent this issue is to use case studies, such as a campus network. A campus is a small-scale community with easily accessible data and more feasible testing of potential changes.

### Bio-inspired network design

Stemming from the success of product-based bio-inspired design, including Velcro and self-cleaning surfaces [24, 25], bio-inspired network design presents a novel strategy for improving the sustainability and resilience of large-scale systems. The approach takes inspiration from ecological food webs, the network of predator-prey interactions making up an ecosystem [26]. Ecological food webs have evolved over millions of years, effectively using all available resources to survive a wide range of disturbances and grow. Past work has shown that designing human networks using the *structure* of food webs can result in sustainability, resilience, and cost improvements. Work drawing inspiration from the *functioning* of food webs has shown improvements in sustainability and resilience [14, 19, 27, 28]. Resilience improvements were found to result from mimicking characteristics related to food web cycling [29] and food web pathway redundancy vs. efficiency [14, 19, 27, 30].

Ecologists study the structure and functioning of ecological food webs using Ecological Network Analysis (ENA). This approach is able to quantify characteristics of these biological networks that are related to their successful and long-term functioning, both during normal operations as well as during disturbances. The quantification of these network characteristics inspired the application of ENA and associated ecosystem behaviors to human networks with similar sustainability and resilience goals. The promising results have suggested both architectural (connectivity) and functional (flow magnitude) design changes for human networks that result in more sustainable and resilient, and more biologically similar, network designs. For example, power grids redesigned to mimic the structure of ecological food webs were found to have improved performance over their traditionally designed counterparts when disturbances took out 1–3 grid components [30]. This approach has also suggested that industrial resource networks can further emissions reductions [9] and reduce consumption of expensive imports such as freshwater [27, 31] if the appropriate characteristic biological network structures are adopted. Industrial symbiosis studies seeking to improve network sustainability have arrived at some principles, such as cyclical flows and reuse, which are similar to those that ENA has identified as being characteristic of biological ecosystems [26, 32]. Additionally, however, engineering investigations of ENA-quantified ecosystem characteristics have indicated novel behaviors that, if adopted in human networks, can support both sustainability *and* resilience goals [33, 34]. This is critical as goals like sustainability and resilience can be difficult to align from a strict engineering design perspective. For example, efficiency improvements will often create a more brittle network. Investigations into ENA for human networks have thus far focused on industrial resource networks [9, 35], water distribution networks, and electric power grids [30], however there remains significant potential that biological networks can offer additional novel approaches for achieving goals such as sustainability and resilience.

Using ENA to consider potential impacts of small changes on a larger network can result in solutions that are easier to implement than other network level solutions, such as net-zero communities. Creating a net-zero community can result in beneficial results for a community as a whole, but requires significant resources and planning. Studies using ENA provide a method of understanding the network impact of individual solutions. Additionally, net-zero projects often focus on energy or carbon emissions, while ENA can be used to consider other network aspects, such as waste or water flows, in conjunction with energy flows. ENA can be used to consider networks of many sizes and both net-zero communities and ENA studies can result in positive system level improvements and can even be used in conjunction for greater impact.

### University campuses as case studies

ENA metrics are here applied to a university campus to discover how modifications affect the sustainability and resilience of the campus to disturbances that may cause, for example, power outages or pipe failures. A campus network case study is used here as a proxy for larger and more complex multi-use net zero communities. NZCs, while of significant interest, pose significant modeling and simulation challenges due to difficulties surrounding data availability. Campus networks on the other hand have many similarities with NZCs. Most large university campuses contain many different networks and buildings that serve a variety of functions, including residential, commercial, dining, sports, agriculture, offices, and transportation. Some campuses, such as that of Texas A&M University (the case study used here), also contain independent utility services that are willing and able to share data and information that may be difficult to acquire from other communities due to proprietary and safety reasons. Accessing information on multiple types of networks (e.g. water, waste, energy) is a significant issue surrounding the study of NZC and industrial networks–industries often classify their inputs and outputs as proprietary information and at a community level power grid information is often protected for national security.

Considering all these different functions and characteristics, some university campuses are residential/commercial/industrial communities very similar to what one would find in a NZC, just at a smaller scale. Residential areas, where students live full time, can provide analogies for larger residential communities or neighborhoods. Dining halls, book stores, on-campus restaurants and convenience stores represent retail institutions. On and off campus transportation networks can include buses, cars, bike routes, pedestrian paths, which mirror those of larger municipalities. Academic administrative buildings can also represent municipal administrative centers. Universities are also often locations of intense activity, where hundreds or thousands of students, faculty and staff participate in classes and extracurricular activities. While academic classes or research are the most evident, campuses are home to a variety of other activities. Some events that commonly occur on the Texas A&M University campus, for example, include sporting events, movie and theater showings, farmers’ markets, community volunteering, and conferences. Beyond participation in classes, students often participate in clubs, work part- or full-time jobs and contribute to a vast range of smaller on-campus events throughout the year. A university campus case study provides a route to model and understand the impact that bio-inspired network designs would have on the sustainability and resilience of larger-scale NZCs.

Proposed changes at a campus level also lack the need for negotiations or proprietary concerns that make implementing industrial symbiosis and NZC changes difficult. Many universities have a single actor in control of their own utilities and operations, as opposed to industrial networks that contain multiple businesses that will each weigh their own interests against the interest of the collective or residential/commercial/industrial NZCs that have even more decision makers and red tape to consider. Universities that are served by outside utility providers often have close ties to local municipalities that can make collaboration easier. University administration may also be more likely to adopt suggested changes at a system level, since the university clearly benefits from both individual and systematic changes that occur on a single campus. As a result, ENA studies and suggestions are more likely to be supported and implemented on a university campus, providing concrete examples for larger scale NZCs and industrial networks who are otherwise reluctant to make changes if their individual benefit is not clear.

Current efforts to improve on campus sustainability fall into a range of different categories. The *Sustainability Tracking*, *Assessment*, *and Rating System* (STARS) is a self-reporting framework that has received reports from 1,004 colleges and universities [36]. STARS rates universities based on five areas: academics, engagement, operations, planning & administration, and innovation & leadership. Within the operations category, energy and water are two of the main reporting subcategories. Energy initiatives are focused on either reducing the energy consumption of buildings or utilizing renewable energy. For example, American University installed a building with passive solar air heating [37], Ball State University required construction of LEED Silver buildings [38], Miami University operates geothermal heat pumps [39], and George Washington University installed a photovoltaic array over a walkway [40]. Water initiatives are focused on water usage and rainwater management; Cornell University is using non-potable water to irrigate athletic fields [41], Pacific University has invested in multiple 10,000 gallon rainwater collection cisterns [42], and Rice University has created seven green roofs [43]. Many of the changes made by universities focus on the building-level rather than the network-level, which misses a host of opportunities for improving the overall campus network. The smaller scale initiatives on campuses have also failed to capture the network level impact of the implemented changes.

The work here uses ENA and inspiration from food webs to better understand how renewable sources of energy and water can be used to improve the resilience of a university campus. The study focuses on the electricity, domestic hot water and domestic cold-water networks on the Texas A&M University main campus. Two common modifications made at the building level, the addition of solar panels and rainwater collection, are investigated for their affect the *network resilience* of the overall campus. These are both common strategies to improve the sustainability of a building and can be considered much easier to implement than major changes to the pre-existing energy or water network of a campus. Understanding how they impact the sustainability and resilience of the overall campus network offers more support for universities to invest in these types of modifications.

## Methods

### Campus network case study–original

The Texas A&M University campus in College Station, Texas was the central case study for this work, defined as the area of the campus north of George Bush Drive and Wellborn Road and south of Texas Avenue. This section of the campus was selected based on the wide range of building functions within the area and the clear system boundaries (three major roadways). This area is known as the “main campus” of Texas A&M University and contains academic buildings, research labs, residential areas, dining halls, sports complexes, and several other building types. Additionally, the main campus includes two utility plants that provide electricity and water primarily to campus buildings. The focus of this study was the utility networks connected to these two plants.

The on-campus utility plants provide electricity, chilled water, heating hot water, and domestic cold and hot water. Since the chilled and heating hot water networks are largely considered closed systems, the electricity and domestic water networks were considered to have higher potential for improvement and the first two (chilled and heating hot water) networks were excluded from the study. All the networks used here were constructed with data from Texas A&M’s Utilities and Energy Services. This included average monthly consumption data for Fall 2018 for all main campus buildings that received utilities from the two on-campus utility plants (see Table 1). Approximately 80% of main campus buildings are included and the excluded 20% had little to no impact.

**Table 1:** Fall 2018 average monthly electricity and domestic water consumption for the functional groups. This does not include utility plants, whose needs are considered self-addressed.

	Average Monthly Consumption for Fall 2018		
Functional Group	Electricity kWh	Domestic Cold Water gallons (m^3^)	Domestic Hot Water gallons (m^3^)
Academic	6343567	5526865 (20921)	355301 (1345)
Administrative/Student Services	1154520	871090 (3297)	9228 (35)
Dining Halls	352155	775239 (2935)	446104 (1689)
Event Venues	20500	26427 (100)	-
Garages	173555	9143 (35)	14 (0.5)
Greenhouses	27468	27725 (105)	-
Health Center	74499	28816 (109)	2359 (9)
Multipurpose	1611734	1369522 (5184)	302309 (1144)
Museum	21828	14401 (55)	-
Residential	2317723	7075009 (26782)	2047981 (7752)
Sports	1654693	1258966 (4766)	-
TV and Radio Station	42480	3521 (13)	-

**Fig 1:**
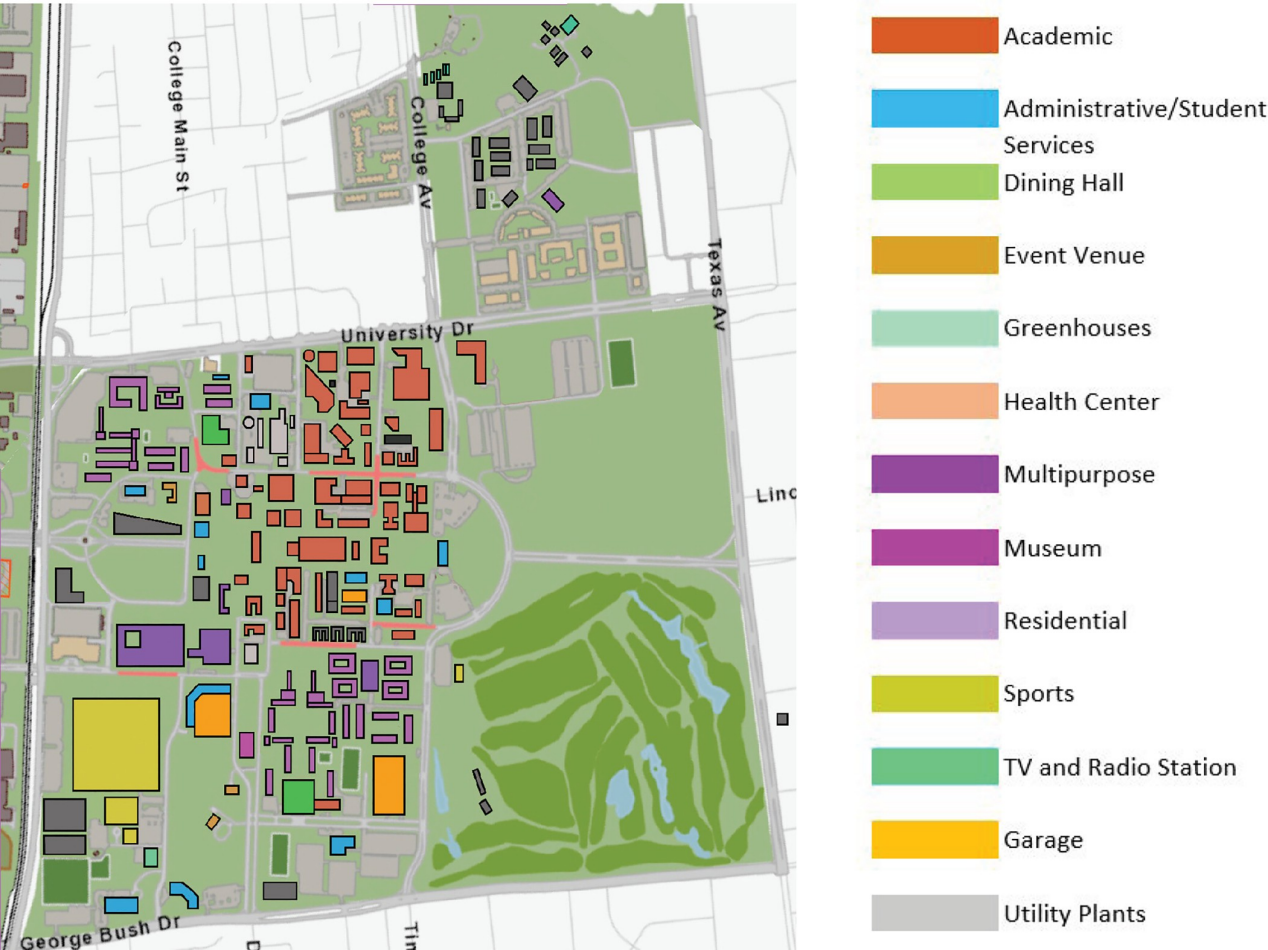
Map of the Texas A&M University main campus (left) showing a color-coded aggregation of the functional groups on the right.

**Fig 2:**
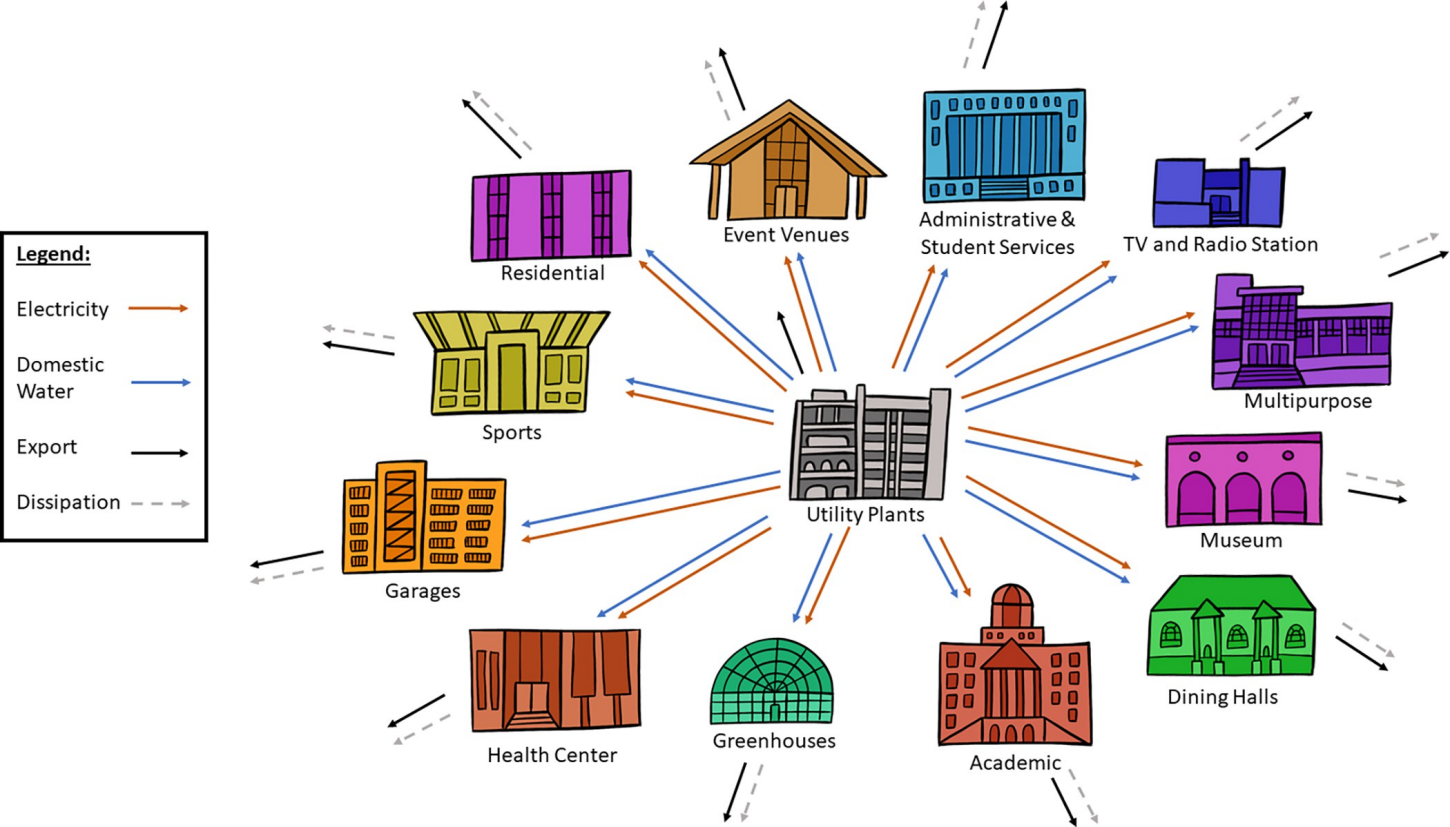
Diagram of the campus network model for electricity and domestic cold water, showing all thirteen actors. The diagram for domestic hot water is the same with the exclusion of the event venues, sports, TV and radio stations, greenhouses, and museum.

The buildings were then aggregated by *function* to make the actors of the network more closely resemble ecological food webs, where each actor in the network represents a species or functional group rather than an individual organism [44, 45]. An individual building in the campus network is analogous to a single organism in a food web and so functional groups for each are groupings made based on the functional role they serve for the network. A total of thirteen functional groups were formed for the campus network (see in Figs 1 and 2): academic buildings, administrative and student service buildings, dining halls, event venues, greenhouses, a health center, multipurpose buildings, a museum, residence halls and apartments, sports facilities, a TV and radio station, garages, and utility plants. The electricity and domestic cold-water networks include all thirteen actors. The domestic hot water network only has eight actors as it does not include the event venues, sports facilities, TV and radio station, greenhouses, or museum.

The campus water and energy networks show that electricity and water are sent from the utility plants to each of the functional groups. Water is then exported to a wastewater treatment plant (off-site) and electricity is dissipated (meaning no value remains after it is used) after reaching the building consumer. These constraints were applied to the campus model and satisfied a node balance requirement (what goes in a node equals what goes out) for the ENA method [46]. The wastewater treatment plant was not considered an actor since it is located off-campus, outside of the system boundaries. The effects of leakages was found to be negligible and so it was neglected in the network models.

### Campus network case study–modifications

Modifications were made to the campus network model to determine their impact on resilience and sustainability efforts. The addition of solar panels and rainwater collection systems were tested, the former affects the electricity network and the latter affects both domestic water networks. Both are commonly documented strategies in the STARS database [36]. Other modifications, such as creating a new greywater reuse system, were not chosen as they require major changes (infrastructure) that were not feasible.

**Fig 3:**
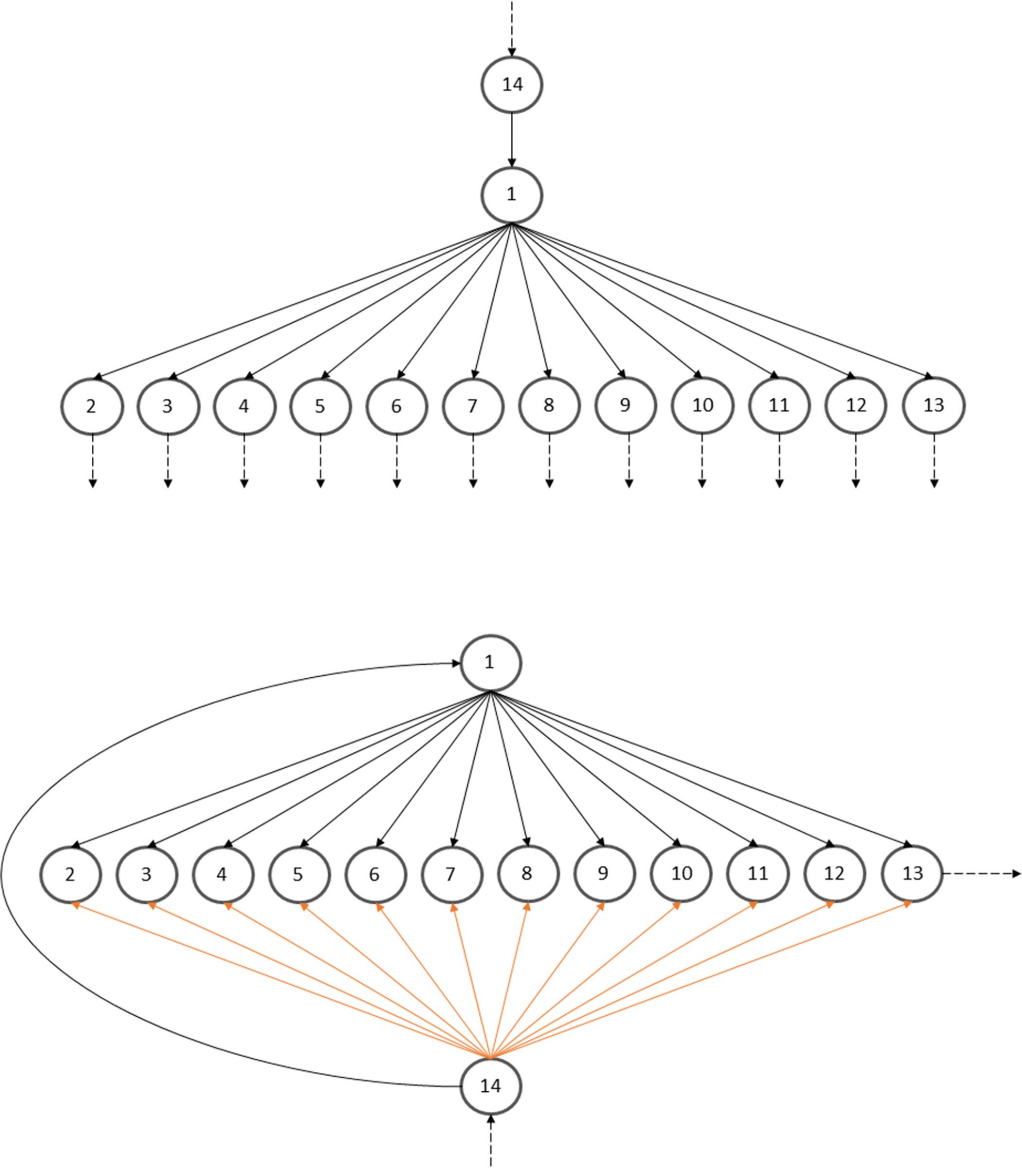
Digraph for the electricity and domestic cold-water networks. The top digraph represents the original electricity and domestic cold-water network while the bottom digraph represents the modified network. The digraph for the domestic hot water network is the same, but with only nine nodes. Node 1 represents the utility plants. Node 14 represents the environment. Nodes 2–13 represent the other actors. Orange arrows represent the added links.

Directional graphs, or digraphs, of the original and modified networks are shown in Fig 3. Each node represents a functional actor (one of the 13 indicated in Figs 1 and 2) and each arrow represents the directional flow of either electricity or water. The environment has been added as an additional actor to the structural networks in order to capture flows to and from the environment.

The amount of electricity or water passing through each of the new links (added for solar panels or rainwater collection and use) was determined based on percentages of the total required amount. Magnitudes were tested from 0–100% in 1% increments for all three network types (electricity, domestic cold water, and domestic hot water). For example, one iteration tested the scenario where each functional group had solar panels that generated 10% of their required electricity, reducing the amount received from the utility plants to 90%. This method was chosen to reveal overall trends associated with making these types of modifications.

### Ecological metrics–structural

Ecological Network Analysis (ENA) was originally created based on information/graph theory to quantify interactions within ecological food webs (the predator-prey interactions within an ecosystem) [47, 48]. Many ENA metrics overlap with metrics from Social Network Analysis (SNA), which is also built from information theory but focuses on quantifying social patterns and interactions in a network [49, 50]. ENA is used by ecologists to explore predator-prey interactions in a network, while SNA is used to study interactions between individuals and/or organizations.

**Fig 4:**
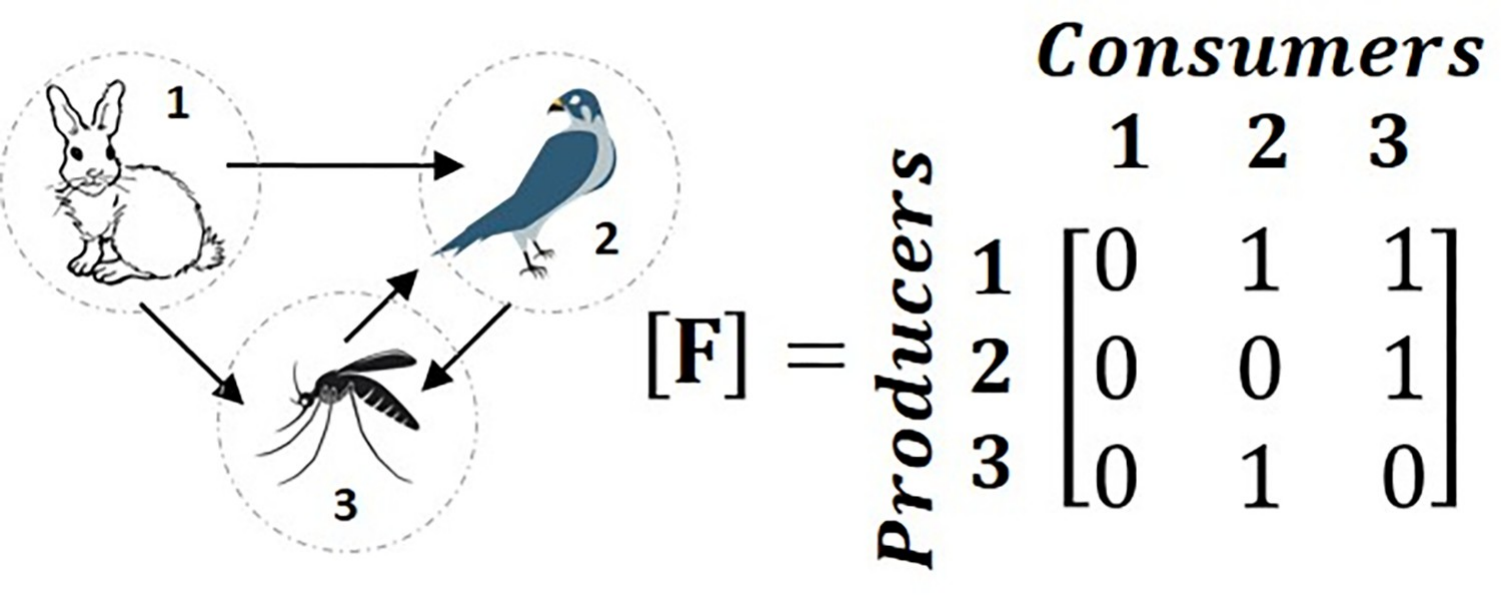
Structural matrix for a hypothetical food web, used with permission from [51]. Flows are transferred from the row to the column. For example, energy is transferred from the rabbit (actor 1) to the bird (actor 2) so there is a one in row 1 and column 2.

Ecologists use digraphs, such as the two shown in Fig 3 above, to visually document the predator-prey relationships within ecological food webs. Each node represents an actor and each arrow represents the transfer of materials and energy. Once the digraph of a network is constructed, the network structure is quantified using a matrix from which metrics quantify the behavior of the network. There are two different types of matrices and metric groups: structural and flow. Structural metrics only require knowledge of the network *structure*, quantified in a structural matrix [**F**] (a hypothetical digraph to matrix example is shown in Fig 4). Flows moves from the producers (rows) to the consumers (columns) such that the presence of a connection from actor 1 to actor 2 (actor 2 consumes actor 1) is documented as a 1 in the *f*_*ij*_ entry for *i* = 1 and *j* = 2. Where there is no connection the entries are zeros.

The size of a network can be described by the number of actors within the network (*N*). The total number of *links* (*ties* in SNA) in the network (*L*) can be found by summing the total number of non-zero values in [**F**] [32]. Linkage density (*L*_*D*_) is the total number of links in the network divided by the total number of actors. The prey to predator ratio (*P*_*R*_) is the ratio of the number of producers or prey (*n*_*prey*_) to the number of consumers or predators (*n*_*predator*_) [32].

The specialized prey fraction (*P*_*S-prey*_, Eq 1) is defined as the average number of prey connected to only one predator, while the specialized predator fraction (*P*_*S*_, Eq 2) is the average number of predators connected to only one prey [32]. High values for either of these metrics can indicate that the network will have more issues recovering in the case of a disturbance. Generalization (*G*, Eq 3) indicates the average number of producers that a consumer will interact with in the network, or how much variety a consumer has in selecting a producer, and is calculated by dividing the number of links by the number of predators [32, 52]. Generalization for ecosystems represents the average number of prey that an average predator will interact with. A high value for generalization indicates that consumers in the network have connections with a larger number of producers, which indicates the consumer will have a greater chance of recovering if a single producer is removed. Cyclicity (*λ*_*max*_, also known as *eigenvalue centrality* in SNA) measures the strength and presence of cycling within the network. It is defined as the maximum real eigenvalue of the transpose of [**F**] [32, 53]. A cyclicity value of zero indicates no cycling within the network. A value of one indicates the presence of one basic cycle and any value greater than one indicates an increasing number and complexity of cycles [53].


$$P_{S-prey}=\frac{\sum_{j=1}^{N}\{\begin{array}{c} 1\;for\;\sum_{i=1}^{N}F_{ij}=1\\ 0\;for\;\sum_{i=1}^{N}F_{ij}=0 \end{array}}{count\;non-zero\;rows\;[F]}$$ (1)

$$P_{S}=\frac{\sum_{i=1}^{N}\{\begin{array}{c} 1\;for\;\sum_{j=1}^{N}F_{ij}=1\\ 0\;for\;\sum_{j=1}^{N}F_{ij}=0 \end{array}}{count\;non-zero\;columns\;[F]}$$ (2)

$$G=\frac{L}{n_{preadtors}}$$ (3)

$$\left\{ \begin{array}{c} \lambda_{max}=maximum\;real\;\lambda \;of\\ |F^{-1}-\lambda I|=0 \end{array}\right.$$ (4)

High specialized predator and prey fractions (Eqs 1 and 2) have been linked to networks being more susceptible to disturbances [27, 54]. These metrics have the potential to indicate whether campus modifications are able to improve resilience. Generalization has been used in the past to find that EIPs have very few companies acting as a source of materials in the network [26]. As a result, the generalization value tends to be lower in EIPs than food webs. We can expect a similar generalization value for the current campus network as the utility plant functional group is the only source in the network. The modifications may shift the generalization values to be closer to that of food webs. A higher value for cyclicity can indicate that a network effectively utilizes and retains materials or energy within the network.

### Ecological metrics–flow

**Fig 5:**
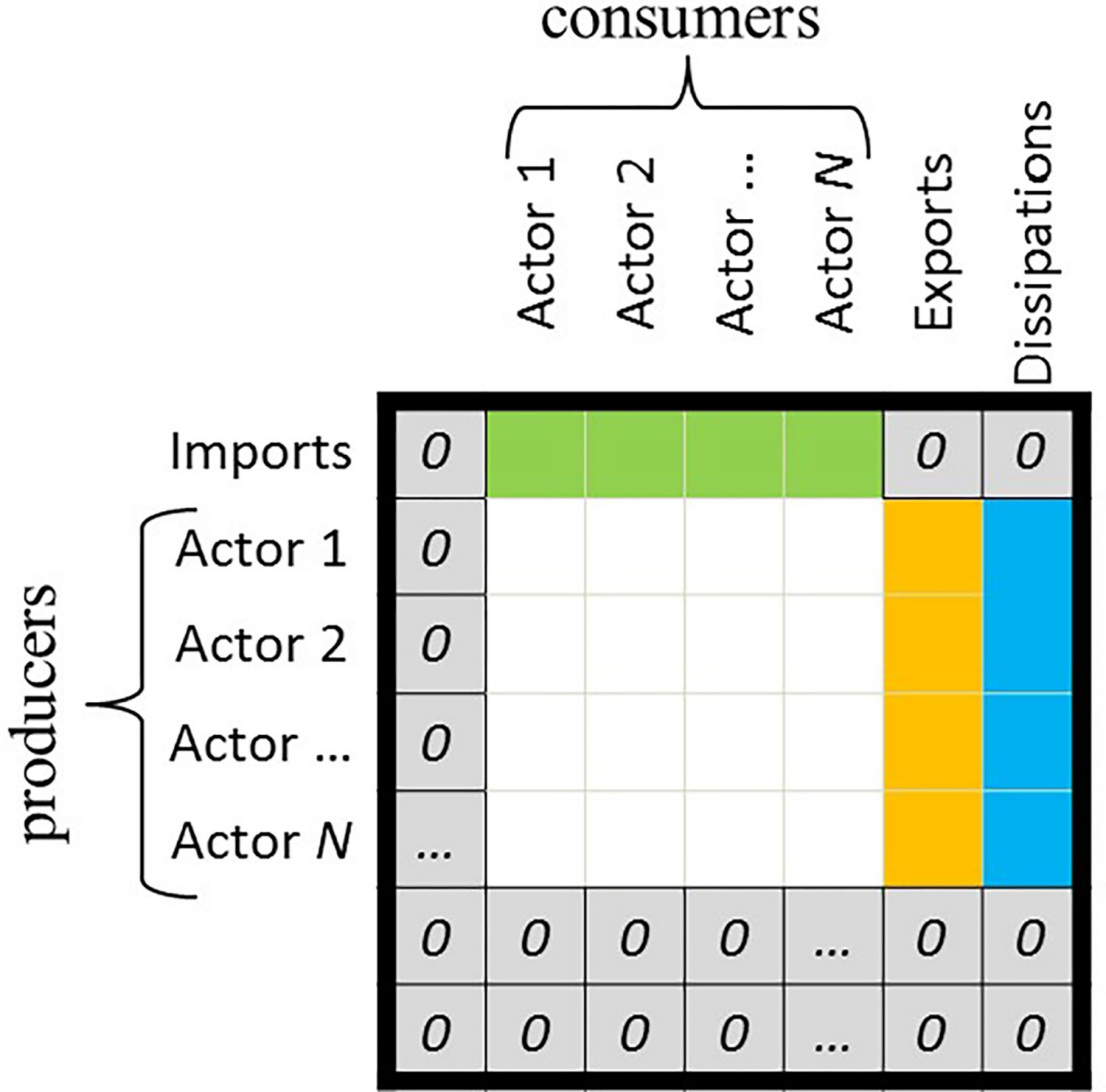
The ecological flow matrix [T], used with permission from [55]. Flows are transferred from row to column. The first row is system imports and the last two columns are system exports (useful and dissipation).

Flow metrics require additional knowledge about the *magnitude* of the flows within the network as well as imports, exports, and dissipations to and from the system. This information is described using an (*N*+3) x (*N*+3) flow matrix [**T**] as shown in Fig 5. Imports are entered in row zero, exports are entered into row *N*+1 and dissipations (non-useful exports) to the environment are entered into row *N*+2. Although more data intensive, metrics calculated from the flow matrix describe the functions of the network in more detail, including the efficiency and redundancy of flows, the network’s robustness to disturbances, and the percent of total flow that is cycled within the network before leaving.

The magnitude of flows passing through the entire network is defined as total system throughput (*TST*_*P*_) and is the sum of [**T**]. This defines the total amount of material moving through the system and can represent the size and level of activity within the network. Ascendency (*ASC*, Eq 5) measures the amount of constraints on the system. The higher the *ASC* the more constrained the flows are within the network, or the fewer pathways there are to get from A to B leading it to be representative of pathway efficiency in the network. Development capacity (*DC*, Eq 6) is the upper bound to *ASC* [56] and quantifies the number of network connections that can still be further developed, identifying the amount of pathway redundancy remaining. Ecological robustness (*R*_*ECO*_, Eq 7) is dependent on the balance between *ASC* and *DC*, also known as the degree of system order [57], and is maximum when *ASC/DC* = 1/*e* and is zero when *ASC/DC* is either one or zero [27, 57]. Ecologists have shown that systems in nature appear to have a unique balance between pathway efficiency and redundancy that enables them to withstand disturbances, resulting in a maximum *R*_*ECO*_. A study of ecosystems found that they tended to have a range of I that resulted in peak *R*_*ECO*_, an area called the “Window of Vitality” [56, 57]. The ecological robustness metric can potentially be used to find the ideal percentage of solar power or rainwater collection per functional group to increase the resilience of the campus networks to outside disturbances, such as power outages or major pipeline leaks.


$$ASC=\sum_{i=1}^{N+3}\sum_{j=1}^{N+3}T_{ij}\times {log}_{2}(\frac{T_{ij}\times {TST}_{p}}{\sum_{j=1}^{N+3}T_{ij}\times \sum_{i=1}^{N+3}T_{ij}})$$ (5)

$$DC=-{TST}_{p}\times \sum_{i=1}^{N+3}\sum_{j=1}^{N+3}\frac{T_{ij}}{{TST}_{p}}\times {log}_{2}(\frac{T_{ij}}{{TST}_{p}})$$ (6)

$$R_{ECO}=-1\times (\frac{ASC}{DC})\times ln(\frac{ASC}{DC})$$ (7)

## Results

Table 2 shows the ENA metric results for all the network configurations along with the average values for ecological food webs. All food web data was gathered from the free R program *enaR* [47]. The first four metrics (specialized prey fraction, specialized predator fraction, generalization and cyclicity) are all structural metrics that do not depend on the magnitude of the flows within the network. As such, the metric values are equal for all the modified networks, except 100%, since the structure of the network itself does not change in the partial modifications. The last column is the flow metric robustness, which does change due to magnitude changes and is therefore different for every case. In all cases, the generalization value for the campus networks increased for the modified networks. The electricity and domestic cold-water network value increase from 6.5 to 12.5 while the domestic hot water network increases from 4 to 7.5. The cyclicity value is zero for both the original networks and all modified networks, indicating that none of the networks experience cycling. Contrarily, ecological food webs have an average value of 6.64 and display much higher levels of cycling. Both the specialized prey fraction and specialized predator fraction decreased after the modifications.

**Table 2:** ENA metrics for all networks. Modifications for electricity and domestic water represent the percentage of solar power and rainwater collection respectively. Food web values are taken from [46, 55].

Network Type	Modification	ENA Metrics				
		*P*_*S-prey*_	*P*_*S*_	*G*	*λ*_*max*_	*R*_*ECO*_
**Electricity**	*None*	*0*.*077*	*1*	*6*.*5*	*0*	*0*.*344*
	10%	0	0.077	12.5	0	0.363
	20%					0.368
	30%					0.367
	40%					0.364
	50%					0.361
	60%					0.357
	70%					0.355
	80%					0.354
	90%					0.355
	100%	0	1	12	0	0.362
**Domestic Cold Water**	*None*	*0*.*077*	*1*	*6*.*5*	*0*	*0*.*337*
	10%	0	0.077	12.5	0	0.360
	20%					0.367
	30%					0.368
	40%					0.366
	50%					0.363
	60%					0.360
	70%					0.358
	80%					0.357
	90%					0.359
	100%	0	1	12	0	0.365
**Domestic Hot Water**	*None*	*0*.*125*	*1*	*4*	*0*	*0*.*301*
	10%	0	0.125	7.5	0	0.344
	20%					0.359
	30%					0.365
	40%					0.368
	50%					0.368
	60%					0.367
	70%					0.366
	80%					0.366
	90%					0.367
	100%	0	1	7	0	0.367
**Food Webs**	*-*	*0*.*14*	*0*.*10*	*9*.*69*	*6*.*64*	*0*.*361*

**Fig 6:**
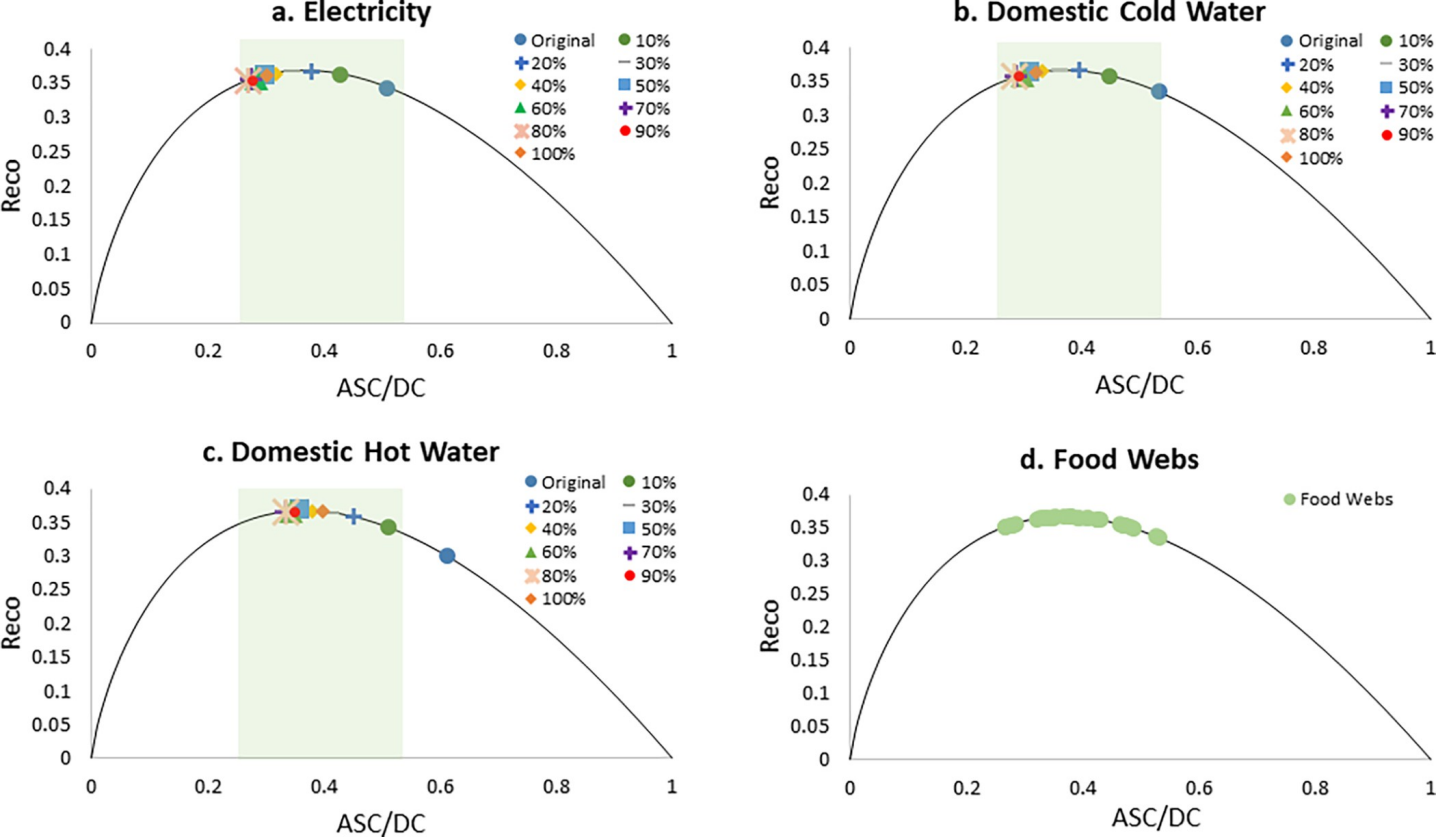
Ecological robustness (*R*_*ECO*_) vs. *ASC/DC*. From top-left to bottom-right: (a) Electricity, (b) Domestic Cold Water, (c) Domestic Hot Water and (d) Food Webs. The shaded green region represents the range of food web values, shown in detail in the bottom-right figure (d). Detailed information on the data is available in S1 Table.

Fig 6 shows all the networks plotted on the robustness curve, including food webs and both the original and modified campus networks. The shaded green region on three of the plots represents the range of the food webs values or the “Window of Vitality.” The robustness plots show that adding solar power and rainwater collection systems to the campus networks does affect the overall robustness of each of the networks. On the robustness plot for the electricity network, as the percentage of solar power increases, the points move from the right side of the curve to the left side until 80%. This signifies an increase in the pathway redundancy of the network. As the percentage increases past 80%, the points move back towards the right, but stop before the peak of the curve. The maximum robustness value is achieved at 23%. The domestic water networks show a similar result. The original networks fall on the right side of the peak. As the percentage of rainwater collection increases for the cold-water network the points move to the left, until 79%, before moving back to the right. The maximum robustness occurs at 27%. The domestic hot water has a turnaround point at 75%. Unlike the other networks it also has two maximums, at 45% and 96%.

## Discussion

Using ENA metrics, both structural and flow, has huge implications for gauging the system-level impacts of sustainability and resilience modifications being considered. Traditional methods of determining sustainability and resilience can be extremely data intensive, requiring detailed emissions knowledge or knowledge of specific system disturbances. The structural metrics here in particular, generalization, specialized prey and predator fractions, and cyclicity, require only knowledge of the presence and direction of interactions. This low bar for their use makes it significantly more likely that decision makers consider the sustainability and resilience of options under consideration.

The increase in generalization (*G*, Eq 3) documented in Table 2 indicates that the consumers in the network are connected to more producers. This is expected, since each of the building functional groups receives water or electricity from more than one source after the modifications. The increase suggests that the modifications will increase the resilience of each of the individual building groups. This is because generalization quantifies the average number of sources any actor can access, resulting in each group having multiple options to meet their needs during a disturbance making it more likely that operations will be sustained even if one or more utility sources become unavailable.

Decreases in both the specialized prey fraction and specialized predator fraction after the modifications reiterate the findings from generalization increasing, indicating that the number of actors who only have one source or sink has decreased. Decreases in these metrics for food webs indicate that the number of prey and predators who have special requirements, only being able to feed off of or be consumed by one other species, has decreased. The decrease in specialized predator fraction for this campus network confirms that the environment, which was a specialized actor in the original network, is now directly connected to more actors than just the utility network. The specialized prey fraction decreased to zero in all cases, indicating that none of the building groups are only connected to one energy or water source after the modifications. An actor connected to only one prey or producer is vulnerable in a disturbance as they are dependent on the survival of a single source. Similar to generalization, the decrease indicates that the number of utility options on average for the system actors has increased. Additionally, the decrease in specialized prey fraction confirms that the functioning of the network has become less dependent on a single source (the utility network) and is therefore less vulnerable if a single key actor is impacted.

The cyclicity values remaining at zero for all of the campus networks was expected as the addition of solar panels and rainwater collection still only provides one other source and does not generate additional cycles (neither modification uses “waste” or byproducts produced in the network). As such, increases in sustainability and resilience for this case study are primarily attributed to the diversification of utility sources for each actor group rather than increases in recycling or reuse in the system (what normally increases in-network cycling).

The results for ecological robustness values support the conclusion that relying on a single source is not supportive of resilience. All three of the networks have at least one maximum robustness value when less than 50% of the needed utilities comes from solar panels or rainwater collection. This indicates that having 100% of a functional group’s utility come from a renewable source may not be the best option in terms of resilience.

These results can be explained by considering potential network disturbances. Assuming that one actor of the electricity network receives 75% of its electricity from the utility plant and 25% from its own solar panels, if the connection to the solar panels is severed, the building will still receive 75% of its needed electricity from the utility plant. If the connection to the utility plant is severed, the functional group will receive 25% of it is needed electricity from the solar panels. In both cases, the functional group will not be able to receive 100% of its needed electricity but would potentially have enough to supply electricity for certain essential purposes.

This also explains why the points move back towards the right side of the curve around 70 to 80%, since the same situation will arise, except with the solar panels providing most of the energy or water. However, the electricity and domestic cold-water networks do not have a second maximum, indicating a preference that the utility plants provide the majority of the utilities. This was likely caused by the structure of the networks. The utility plant is connected to all other actors in the network. If it is removed, all of the functional group actors would need to import their needed utilities from the environment. Thus, it is more pathway efficient to depend more on the utility plant.

The domestic hot water network has two maximums. The main difference between the electricity and domestic cold-water networks and the domestic hot water network was the number of actors or functional groups. The hot water network has eight actors, while the other two have thirteen. While more data would need to be tested to confirm this is the cause, if true, it indicates that smaller campus networks with fewer functional groups can have higher amounts of solar energy generation or rainwater collection and still gain the maximum possible robustness.

When considering carbon emissions, the ideal scenario would be to have 100% of the electricity or water come from a renewable source, which conflicts with the results above. This indicates a potential tradeoff between resilience and carbon emissions. Campuses that are prone to having more severe disturbances, such as natural disasters that interfere with utility services, may want to prioritize a more resilient network. These results suggest that campuses not prone to disturbances may choose to sacrifice robustness for a more carbon neutral network. One potential solution to this tradeoff is to have multiple renewable energy sources. For instance, if 75% of the electricity came from wind energy instead of the utility plant, a similar robustness could be achieved while still staying 100% renewable.

The feasibility of making these types of modifications also needs to be considered. Rainwater collection systems are heavily dependent on the amount rain an area receives, as well as the available space for the collection system itself. The level of rainfall in College Station, Texas varies throughout the year and there are times when supplying 100% of demand through rainwater may not be possible. Similarly, supplying 100% of electricity from solar panels is dependent on the amount of sunlight and space for solar arrays. Additionally, attempting to make both systems entirely renewable is likely a large investment in both time and cost. As such, it may be more feasible for the university to first attempt smaller modifications.

### Limitations and future work

One area of interest that was not discussed here is the waste network on Texas A&M’s campus. Texas A&M Utility Services manages the waste and recycling streams on-campus in addition to the water and energy. A similar study on waste streams may reveal potential opportunities for reuse or increased cycling, and therefore sustainability and resilience, on-campus. One example of an existing initiative on the Texas A&M campus is the Howdy Farm, which encourages students and faculty to drop off compostable waste that is then used to grow produce sold back to students and faculty. Utility Services does not currently collect data on the amount or composition of the materials leaving the campus in these streams making it difficult to propose potential changes. A limitation of any work seeking to follow a waste network in a similar fashion as was done here are the additional regulations surrounding materials labeled as waste. These limitations can make tracking and reuse efforts difficult.

Another limitation in this work was the exclusion of imported utilities to the Texas A&M campus. When problems occur with the on-campus utility plants, electricity and water *can* be provided by off-campus, local utility plants. Conversely, if the surrounding community experiences major disturbances, the Texas A&M utility plants can provide additional electricity to the community. This was not included in the study as this would only occur in the case of an extreme and unlikely disturbance. There was also no available information about the magnitude of these potential flows. These flows however may be worth considering in future work exploring the effects of specific large-scale disturbances on the network.

It is also worth noting that a college campus network could be considered a specialized network. While campus networks often have many of the same components of city or community system, it is often easier to make changes to such components on a college campus. More stakeholders are involved in changes to more complex systems. Despite these differences, the results give insights into changes that a larger scale community might be interested in if they are trying to achieve a goal such as net-zero.

Future work on water networks should also consider greenspaces, such as golf courses or park areas that may be able to utilize graywater. Current consumption data for these areas was not available during this study, so they were excluded. However, the current Texas A&M campus greenspaces do not utilize greywater for their sprinkler systems. Future work must also be done to investigate other university campuses to test for similar results, especially campuses that already have a large solar or rainwater collection presence. A comparison of results between different types of campuses would present an interesting and informative study of the impacts of factors such as the size of the network (small, medium or large campus), location (rural, town, city, etc.), and the number of functional groups. Another differentiating factor is the campus’s relationship with its surrounding area. The Texas A&M main campus has very clear boundaries but other universities (especially those located in the middle of large cities) may be more integrated with their surroundings. Such cases may make it more difficult to gather data, but also hold the potential for a campus to have a positive impact on the resilience of the local utility network. In such cases, the university campus could be considered a segment of a larger utility network.

### Applications

While the specific numerical results demonstrated here may not be accurate to other university campuses, the general findings are applicable. Many university campuses contain similar functional groups to the Texas A&M campus and receive utilities from a single source, whether it is on- or off-site. As more universities transition to renewable sources of water and energy, the resilience implications of those changes must be carefully considered in addition to the sustainability benefits. The analysis done here supports the use of a diversity of utility sources in conjunction with sustainability changes. Net zero communities, cities, and industries that are looking at incorporating sustainability motivated changes must also consider the resilience impacts of those changes. The bio-inspired results highlight options that can address both sustainability and resilience goals. The results also highlight a pitfall of one option, switching to a renewable energy source, which many networks may consider: just swapping one energy source for another still leaves the network vulnerable to disturbances. The results here suggest that a gradual transition to more than one renewable energy source is highly desirable when both sustainability and resilience needs are considered. Additionally, when only two energy sources are available, the results suggest that an ideal configuration is for the network to receive approximately 75% of the needed amount from one source and 25% from the other. For instance, a community investing in renewables might consider using solar for a majority of their required electricity but ensure that they also have a connection to wind or geothermal energy for the remaining 25% to maximize both their network’s sustainability and resilience to unforeseen disturbances.

The results support the ability to use ENA for multi-use networks like net zero communities (NZCs). While the campus network model is smaller scale, the similarities it has with larger scale residential-commercial-industrial NZCs support designers and decision makers to consider these design solutions as viable options. As discussed, results from a campus case study like the one done here can easily (relative to a network of industries or NZC) be implemented to further validate simulation results in future work–hopefully paving the way for their implementation at the larger and more impactful scales of NZCs and industrial networks.

## Supporting information

S1 Table A table of the calculated ecological robustness (R_eco_) and ASC/DC values for all scenarios used to create the plots shown in Fig 6. (XLSX)

S2 Table A table showing the flow matrix [T] for the original, unmodified campus electricity network. (XLSX)

S3 Table A table showing the flow matrix [T] for the original, unmodified campus domestic cold-water network. (XLSX)

S4 Table A table showing the flow matrix [T] for the original, unmodified campus domestic hot water network. (XLSX)
